# Essential Oils and Their Major Components: An Updated Review on Antimicrobial Activities, Mechanism of Action and Their Potential Application in the Food Industry

**DOI:** 10.3390/foods11030464

**Published:** 2022-02-04

**Authors:** Manasweeta Angane, Simon Swift, Kang Huang, Christine A. Butts, Siew Young Quek

**Affiliations:** 1 Food Science, School of Chemical Sciences, The University of Auckland, Auckland 1010, New Zealand; mang207@aucklanduni.ac.nz (M.A.); kang.huang@auckland.ac.nz (K.H.); 2 Faculty of Medical and Health Sciences, School of Medical Sciences, The University of Auckland, Auckland 1010, New Zealand; s.swift@auckland.ac.nz; 3 The New Zealand Institute for Plant & Food Research Limited, Palmerston North 4442, New Zealand; chrissie.butts@plantandfood.co.nz; 4 Riddet Institute, New Zealand Centre of Research Excellence for Food Research, Palmerston North 4474, New Zealand

**Keywords:** essential oil, peel, antibacterial, antimicrobial, mechanism of action, preservation

## Abstract

A novel alternative to synthetic preservatives is the use of natural products such as essential oil (EO) as a natural food-grade preservative. EOs are Generally Recognized as Safe (GRAS), so they could be considered an alternative way to increase the shelf-life of highly perishable food products by impeding the proliferation of food-borne pathogens. The mounting interest within the food industry and consumer preference for “natural” and “safe” products means that scientific evidence on plant-derived essential oils (EOs) needs to be examined in-depth, including the underlying mechanisms of action. Understanding the mechanism of action that individual components of EO exert on the cell is imperative to design strategies to eradicate food-borne pathogens. Results from published works showed that most EOs are more active against Gram-positive bacteria than Gram-negative bacteria due to the difference in the cell wall structure. In addition, the application of EOs at a commercial scale has been minimal, as their flavour and odour could be imparted to food. This review provides a comprehensive summary of the research carried out on EOs, emphasizing the antibacterial activity of fruit peel EOs, and the antibacterial mechanism of action of the individual components of EOs. A brief outline of recent contributions of EOs in the food matrix is highlighted. The findings from the literature have been encouraging, and further research is recommended to develop strategies for the application of EO at an industrial scale.

## 1. Introduction

Antimicrobial agents used to kill or inhibit the growth of pathogenic or food spoilage bacteria can exist in natural or synthetic forms. The use of synthetic antimicrobial compounds as food preservatives has raised consumers’ concerns, since they present numerous toxicological difficulties and may not be safe for human consumption [1]. Hence, over the last two decades, natural antimicrobial agents such as essential oils (EOs) have received renewed interest from the scientific community, owing to their unique physicochemical properties and diverse biological activities [2]. In the definition coined by Rios [3], EOs are aromatic, oil-like volatile substances present in plant materials such as fruits, bark, seeds, pulp, peel, root and whole plant. These substances form in the cytoplasm, and generally exist as tiny droplets sandwiched between the cells. In recent years, increasing awareness about the “green, safe and clean” environment and a growing appeal for “green consumerism” have prompted the production of foods free of synthetic preservatives [4,5].

EOs have been used for medicinal purposes and as therapeutic agents since ancient times [6]. Although food industries utilize EOs as a flavoring agent, their potential as a natural food grade preservative has not been fully explored. EOs present a valuable tool for food preservation due to their natural antimicrobial properties [7]. However, a detailed understanding regarding individual components of EOs, their antibacterial properties, mechanism of action and target organisms is required to support the implementation of EOs as food preservatives. Calo et al. [8] reported that EOs comprise numerous compounds such as aromatic hydrocarbons, terpene (monoterpenes and sesquiterpenes), terpenoids, esters, alcohols, acids, aldehydes and ketones, and their antibacterial activity is not solely contributed by any one compound. Recognizing the most potent antibacterial compounds from EOs is often tricky due to their chemistry complexity. To date, most studies have focused on studying the antimicrobial activity of EOs [5,8], with little discussion on the antibacterial activity of individual components in the EO or their mechanism of action. The antibacterial activity of EOs is not reliant on one specific mode of action; instead, EOs can attack several targets in a cell to inactivate the bacterium [7]. Evaluating EO’s antibacterial properties and mechanism of action of their components may provide new insights into their applications in the food industry. This approach may reveal the concealed antibacterial properties of individual EO components, otherwise masked when EOs are studied as one single substance.

Several reviews [2,9,10] have outlined the antimicrobial activity of EOs extracted from various plant sources such as stem, bark, leaf, fruit, and seeds, but did not discuss the waste parts such as peel. The amount of waste produced by fruit processing industries is diverse [11]. Fruit peels generated by food industries are treated as agro-waste and are discarded in landfills, composted or fed to livestock [12]. Fruit waste produced in enormous quantities during commercial processing could present severe environmental threats [13]. Ayala-Zavala et al. [14] proposed using fruit by-products as an antimicrobial food additive, reporting that mandarins, papayas, pineapple, and mangoes accounted for 16.05%, 8.47%, 13.48% 11% of peel waste, respectively. On the other hand, fruit peel is a rich source of EOs and contains promising novel components of potential pharmacological, pharmaceutical and economic significance [13]. Moreover, fruit peel EOs are classified as GRAS (generally recognized as safe) and can be used to improve food safety due to their unique antimicrobial properties [15].

Studies on EOs extracted from various plant sources are well represented in the literature, and it is widely recognized that EOs possess a range of biological activities. For instance, EOs extracted from thyme [16], oregano, lavender [4,17], cinnamon, clove [18] and turmeric [19] have antibacterial, antifungal, algicidal, antioxidant, anticancer and anti-inflammatory activities. Chemical compositions and biological properties of plant EOs, in general, have been discussed in detail in reviews by Bakkali et al. [20], Burt [5] and Ju et al. [21]. A substantial amount of work has been carried out to evaluate the antimicrobial properties of EOs extracted from fruit peels; however, none of the reviews in the compiled data have exclusively discussed peel EOs. In light of these factors, this review aims to summarize the most significant findings of the antimicrobial properties of fruit peel EOs and their major components that contribute to microbial inactivation, with a focus on the mode of action of EO/EOs components. Finally, the application of various plant-derived EOs in the food industry is discussed, and future research directions and applications are presented.

## 2. Chemical Composition of Fruit Peel Essential Oils

**Figure 1:**
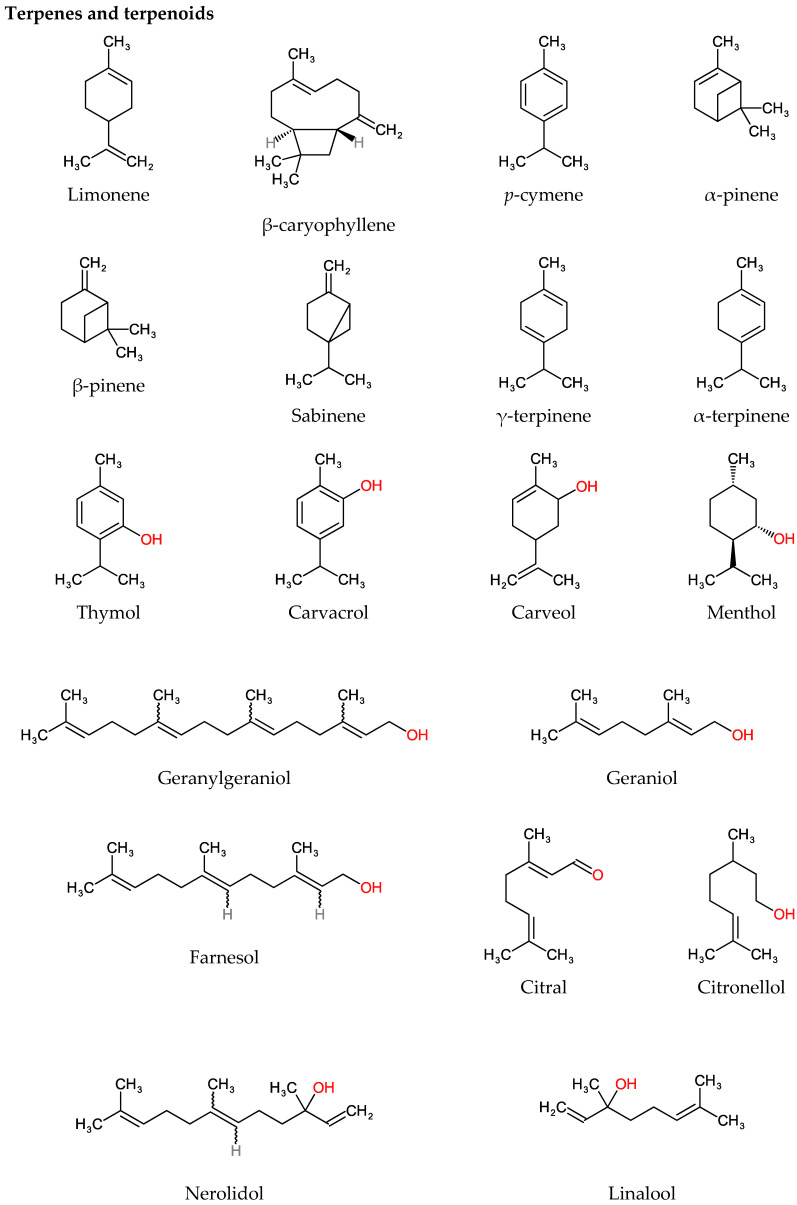

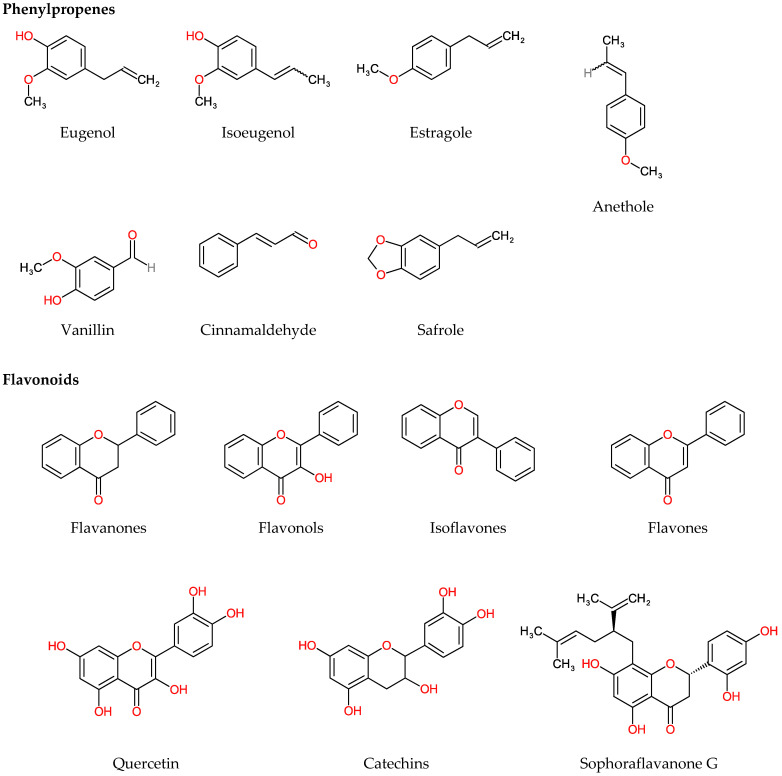
Chemical composition of essential oils (EOs).

Plants produce a variety of chemical compounds with antimicrobial properties. Some of these compounds are always present, while others are secreted in response to stress, such as infection, damage, predators, and weather variations. The chemical constituents in EOs are prone to variations depending on the time of harvest, cultivar, and the extraction method. Hydro distillation and steam distillation are frequently used to produce EOs at a commercial scale [5]. Identifying the most active compounds from EO can be a cumbersome process. Gas chromatography (GC), gas chromatography-mass spectrometry (GC-MS) [22,23,24], high-performance liquid chromatography (HPLC) [25,26,27] and liquid chromatography coupled to mass spectrometry (LC-MS) [28] are the most widely used methods to study the chemical composition of EOs. The primary chemical components of EOs are terpenes and polyphenols. Figure 1 shows the structural formula of some of the major components of EOs. These chemical compounds have been reported to have antimicrobial properties and their mechanisms of action are discussed later (Section 4).

Terpenes can be defined as a framework of numerous isoprene units (C_5_H_8_) merging to form a hydrocarbon molecule. They are derived from mevalonate and mevalonate-independent pathways [29]. Terpenes usually exist in EOs in the form of monoterpenes (C_10_H_16_) or sesquiterpenes (C_15_H_24_). However, other long-chain molecules such as diterpenes (C_20_H_32_), triterpenes (C_30_H_48_), tetraterpenes (C_40_ H_64_) are found in EOs in minor quantities [30]. Examples of terpene compounds include β-caryophyllene, *p*-cymene, α-pinene, β-pinene, limonene, sabinene, γ-terpinene, α-terpinene, β-myrcene, cinnamyl alcohol, and δ-3-carene. Additionally present are terpenoids, identified as an oxygenated derivative of terpene compounds with an additional oxygen molecule, or their methyl group being moved or eliminated. Terpenoids are further categorized into esters, aldehydes, ketones, alcohols, ethers, and epoxides, with examples including menthol, geraniol, eugenol, thymol, carvacrol, geraniol, linalyl acetate, linalool, citronellal, citronellol and terpineol [7,31].

Polyphenols are secondary metabolites widely distributed in nature, usually derived from the phenylpropanoid pathway [32]. Polyphenols can be categorized into phenylpropenes and flavonoids, based on the number of phenol rings [33]. Phenylpropenes have derived their name from the six-carbon aromatic phenol group, and the three-carbon propene tail of cinnamic acid formed during the first step of phenylpropanoid biosynthesis [34]. Flavonoids are a group of phenolic compounds with a carbon framework (C_6_-C_3_-C_6_). The basic skeletal structure of flavonoids comprises a 2-phenyl-benzo-γ-pyrone consisting of two benzene rings (ring A and ring B) cross-linked to a heterocyclic pyrone (ring C) [35]. Based on the degree of oxidation, flavonoids are further classified into flavones, flavonols, flavanones and others [36].

A detailed analysis of the EOs of orange peel identified an abundant amount of limonene, ranging between 73.9–97.6%, while other monoterpenic alcohols, namely geraniol, linalool, nerol and α-terpineol, were present in minor quantities at concentrations of 2.1%, 4.1%, 1.5%, 2.4%, respectively [24]. This finding was in agreement with Ambrosio et al. [22] and Guo et al. [37], who reported similar compounds in orange peel EOs. However, some compounds such as cis-*p*-mentha and trans-*p*-mentha [22,37] were not reported previously [24]. These differences could be attributed to the different cultivars or growing conditions of the fruit analyzed in these studies. Moreover, a close resemblance was noted in the limonene content of grapefruit peel EO, which was present at a concentration of 93.3% [23], 91.5% [38] and 91.8% [39]. Other monoterpene compounds such as β-myrcene, α-pinene, sabinene, linalool and thujene were also reported [23,38,39]. In pummelo peel EO, limonene contributed up to 55.7% of the total EO composition, followed by β-pinene (14.7%), linalool (6.2%), β-citral (4.1%), germacrene-D (2.7%), α-pinene (2.3%), α-terpineol (2.0%), geraniol (1.6%), sabinene (1.3%) [39]. Tao et al. [40] reported similar compounds but at a much lower concentration, ranging from 0.08% to 0.63%. The difference in the extraction method, such as using a rotary evaporator at 40 °C [38], could have contributed to the significant loss of highly volatile compounds from the EO. Furthermore, Hosni et al. [41] and Hou et al. [42] found limonene to be the main component in mandarin peel EO, but other secondary compounds such as lauric acid, 1-methyl-1,4-cyclohexadiene, methyl linoleate, myristic acid, palmitic acid and β-myrcene were reported only by Hou et al. [42]. More recent evidence [43] highlights that out of 158 compounds found in feijoa peel EO, 89 compounds identified were novel; these compounds include esters, sesquiterpenes, monoterpenes, aromatic hydrocarbons, alcohols, aldehydes, ketones, hydrocarbon, acids and ethers.

Limonene is the predominant component in the EOs of orange [22,24,37,41], grapefruit [23,39], mandarin and pummelo [39,40] peels, and is thought to contribute to most of the antimicrobial activity of the fruit peels reviewed above [44]. However, Ambrosio et al. [22] argued that limonene is present in different concentrations in different fruit peels; thus, the antimicrobial activity of EOs cannot be ascribed solely to limonene. Additionally, studies have reported low antimicrobial activity of limonene when the pure compound was tested [45]. Hence, in citrus fruits, other minor compounds such as α-pinene, sabinene, linalool, β-citral, and germacrene-D could contribute to the antimicrobial activity.

## 3. Antimicrobial Properties of Fruit Peel Essential Oils

The antimicrobial activity of EOs can be seen as the inhibition of cell growth or by cell-killing. However, it is not easy to differentiate between these modes of action. The antimicrobial efficacy of EOs is dependent on their chemical composition, environmental conditions and the structures of the target bacteria (either Gram-positive or Gram-negative bacteria) [46]. Numerous in vitro techniques [47], such as the determination of minimum inhibitory concentration (MIC) and minimum bactericidal concentration (MBC) by broth macro dilution/microdilution or agar disk/well diffusion are applied to determine the efficacy of an antimicrobial compound. Agar disk/well diffusion and broth macro dilution/microdilution are widely used methods in clinical microbiology laboratories [48] and have recently been recognized as useful tools to determine the antimicrobial activity of EOs [49,50].

Many studies have illustrated the antimicrobial effect of fruit peel EOs against drug-resistant, pathogenic and food spoilage bacterial strains. Some studies have found that EOs extracted from the fruit peels of banana [13], pomegranate [1] and citrus fruits such as sweet orange, grapefruit, lime, sweet lemon, mandarin, tangerine and pummelo [22,40,51,52,53] exhibited inhibitory activity against Gram-positive and Gram-negative bacteria. These studies indicate that fruit peels are a potentially valuable anti-microbial resource [42]. A wide range of foodborne pathogens could be inhibited by fruit peel EOs, including *Escherichia coli*, *Enterobacter cloacae*, *Klebsiella pneumoniae*, *Pseudomonas aeruginosa*, *Salmonella enterica* serovar Typhimurium, *Salmonella enteritidis*, *Bacillus subtilis*, *Bacillus cereus*, *Streptococcus faecalis*, *Listeria monocytogenes*, *Proteus vulgaris, Staphylococcus aureus* and others (Table 1). An overview of the antimicrobial activity of various fruit peel EOs and detection methods over the last 15 years is presented in Table 1.

### 3.1. Citrus Essential Oils

Abd-Elwahab et al. [51] reported the efficacy of EOs extracted from citrus peels, i.e., orange, lime, mandarin, and grapefruit, as having moderate to high antibacterial activity against *S. aureus*, *B. subtilis*, *E. faecalis*, *E. coli*, *Neisseria gonorrhoeae* and *P. aeruginosa*. Among those citrus EOs, lime peel EO was the most effective at inhibiting all six strains of pathogenic bacteria. The presence of coumarine and tetrazene in lemon peel [13] and citral, limonene and linalool in other citrus peel EO [51] may have accounted for their antimicrobial activity against these bacteria. On the contrary, Javed et al. [15] reported that amongst all tested citrus peel EOs (mandarin, tangerine, sweet orange, lime, grapefruit) mandarin peel EO possessed the highest antimicrobial activity. The inhibition zone for *Salmonella enterica* serovar Typhi, *E. coli*, *Streptococcus sp.* and *P. fluorescence* ranged from 20 to 30 mm for 10 μL and 9–16 mm for 5μL treatments of mandarin peel EO. The differing concentrations of the citrus peel EOs between the studies might explain these contradictory results.

### 3.2. Orange Essential Oils

Over the past decade, several studies [24,37,53,54,55] have examined the antibacterial properties of sweet orange (*Citrus sinensis*) EO. A broad-spectrum antibacterial activity was observed against a range of foodborne pathogens, confirming its potential to be a natural antimicrobial agent for food preservation. In a study conducted by Guo et al. [37], the antimicrobial activity of cold-pressed and light phase EO extracted from orange peel was compared using *E. coli*, *S. aureus*, and *B. subtilis*. It was reported that light phase EO showed a better antimicrobial activity compared to the cold-pressed EO. The higher antimicrobial activity can be attributed to a higher quantity of carvone and limonene in the light phase EO. Nwachukwu et al. [56] tested the efficacy of orange peel EO extracted using water and ethanol (hot and cold) against *E. coli*, *S. aureus*, and *Bacillus* sp. It was noted that hot ethanol extracted EO was more effective than the water extracted EO at inhibiting the three bacteria strains. Hot ethanol might have facilitated the better release of volatile compounds present in orange peel EO. These findings are similar to those of Ali et al. [55], Bendaha et al. [52], and Kirbaslar et al. [57], who reported similar antimicrobial activity of orange (*Citrus aurantim)* peel EO against *L. monocytogenes*, *S. aureus*, *E. coli*, *E. faecalis*, *B. cereus*, *K. pneumoniae* and *P. aeruginosa*. One of the significant drawbacks of these studies [15,37,51,52,53,55,56,57] was that they fail to consider the MIC and MBC values, thus providing no foundation for EO application in food. However, Geraci et al. [24] and Tao et al. [54] had reported the MIC values of orange peel EO, and as anticipated Gram-positive (*B. cereus*, *B. subtilis*, *S. aureus*) bacteria were reported to be more susceptible to the orange peel EO compared to the Gram-negative (*E. coli* and *P. aeruginosa*) bacteria.

### 3.3. Grapefruit Essential Oils

The antimicrobial activity of grapefruit (*Citrus paradisi*) peel EO against *B. subtilis*, *E. coli*, *S. aureus*, *S. enterica* serovar Typhimurium and *P. aeruginosa* was reported by Deng et al. [23]. It was noted that Gram positive *B. subtilis* was the most sensitive amongst all strains investigated, while Gram negative *P. aeruginosa* was the least sensitive organism. This antibacterial activity may be attributed to the presence of abundant limonene in the grapefruit peel EO [44]. Similarly, pummelo (*Citrus grandis*) peel EO showed good inhibitory activity against Gram-positive bacteria (MIC- 9.38 µL/mL) and moderate activity against Gram-negative bacteria (MIC- 37.50 µL/mL) [40]. Terpene alcohols such as linalool are known for their inhibitory activity against Gram-negative bacteria [58]. Although a substantial amount of linalool was found in the pummelo peel EO, it did not inhibit *E. coli* [40]. This microorganism was only susceptible to pure linalool, but not to EO with linalool as one of the components in a mixture of compounds [59]. The use of EO instead of linalool alone might have contributed towards a higher MIC value of pummelo peel EO against *E. coli*.

### 3.4. Essential Oils from Other Fruit Peels

Several researchers have examined the antibacterial activity of various other fruit peels such as tamarillo [60], bergamot (*Citrus bergamia*) [57,61], sweet lemon (*C. limetta*) [53,62], *C. deliciosa* [63], kumquat (*C. japonica)* [64] and feijoa (*Acca sellowiana*) [65]. Surprisingly, Diep et al. [60] and Mandalari et al. [61] revealed that the tamarillo and bergamot peel flavonoids, respectively, exhibited strong antibacterial activity against Gram-negative bacteria such as *E. coli*, *Pseudomonas putida*, *S. enterica* serovar Typhimurium and *P. aeruginosa*, while Gram-positive bacteria (*B. subtilis*, *L. innocua*, *S. aureus*) were resistant. Similarly, El-Hawary et al. [63] found that *C. deliciosa* EO extracted from its leaves and peel was more effective against Gram-negative bacteria than the Gram-positive bacteria. In contrast, the inhibitions zones for bergamot peel EO (11mm to 16mm) with no clear distinction between Gram-positive and Gram-negative bacteria [57], and sweet lemon EO, demonstrated good antibacterial activity against both Gram-positive and Gram-negative bacteria with inhibition zones measuring between 10 to 35 mm [62].

Due to the difference in their cell wall structure [34], Gram-positive bacteria are more susceptible to EOs than Gram-negative bacteria [23,40,54,66]. However, published data have shown no clear differentiation between Gram-positive and Gram-negative bacteria [60,63]. The reason for this contradictory result is discussed in Section 4. It is somewhat surprising that many studies have assessed the antimicrobial activity by using only the agar disk/well diffusion method [15,22,39,50,51,52,53,55,56,60,63,65,67,68,69,70,71,72]. Agar disk/well diffusion is a quick typing tool used to determine the sensitivity of the bacterial strain. However, this quick typing tool cannot differentiate between bacteriostatic and bactericidal effects. The agar disk/well diffusion is a preliminary method that is not suitable to determine MIC or MBC, since it becomes quite challenging to measure the amount of EO diffused in the medium. Moreover, the hydrophobic nature of EO might pose an added challenge with regard to its ability to diffuse through the media, potentially resulting in uneven distribution. On the other hand, though tedious and time-consuming, broth macro dilution or microdilution methods allow quantifying the exact antimicrobial agent concentration that is effective against the pathogen and visibly distinguishes between bacteriostatic and bactericidal effects [49]. Most of the studies reviewed so far tend to overlook the importance of the broth dilution method for the determination of MIC and MBC of EOs, which is vital for determining the exact concentration required to kill bacteria, a prerequisite for assessing their potential application in food preservation.

**Table 1:** Overview of antimicrobial activities of fruit peel essential oils (EOs) and extracts.

Source of Peel EO	Target Organism	Method Used	Solvent Used	Test Concentration	Remarks	References
Tamarillo	*E. coli*, *P. aeruginosa*, *S. pyogenes*, *S. aureus*	Disk diffusion	MilliQ, n-hexane, ethanol, methanol	115 μL of 100 mg/mL on 13 mm disk	*E. coli* was most sensitive to aqueous extract from the peel (inhibition zone of 24 mm), *P. aeruginosa* was most sensitive to methanol extract.	[60]
Grapefruit	*B. subtilis*, *E. coli*, *S. aureus*, *S. enterica* serovar Typhimurium, *P. aeruginosa*	Disk diffusion, MIC determination	-	20 μL of 100, 50, 25, 12.5, 6.25, 3.125, 1.56, 0.78, 0.39 and 0.195 mg/mL of EO placed on each disk	*B. subtilis* represented a maximum inhibitory zone of 35.59 mm and MIC value of 0.78 μL/mL. *P. aeruginosa* was least sensitive representing an inhibition zone of 8.57 mm and MIC value of 25.0 μL/mL	[23]
Sweet orange, Lemon, Banana	*P. aeruginosa*, *K. pneumoniae*, *Serratia marcescens*, *E. coli*, *P. vulgaris*, *S. enterica* serovar Typhi, *S. aureus*, *E. faecalis*, *L. monocytogenes*, *Aeromonas hydrophila*, *Streptococcus pyogenes*, *Lactobacillus casei*	Agar well diffusion, MIC determination	Distilled water, Methanol, Ethanol, Ethyl acetate	5 mg/mL	*K. pneumoniae* was most susceptible to lemon peel extract (inhibition zone and MIC, 35 mm, and 130 μg/mL, respectively).	[13]
Kumquat	*E. coli*, *S. enterica* serovar Typhimurium, *S. aureus*, *P. aeruginosa*	Disk diffusion and MIC determination by broth microdilution method	Methanol 80%, Ethanol 70%, Acetone, Ethyl acetate, n- Hexane, Chloroform	From 10 mg/mL, 25 μL of extract was placed on each disk.	For all extracts, *E. coli* was most resistant (inhibition zone 11.3 mm and MIC of 679 μg/mL) while *S. aureus* was the most susceptible (inhibition zone 16.7 mm and MIC of 496 μg/mL) strain.	[64]
Sweet orange	enterotoxigenic *E. coli*, *Lactobacillus sp*	Disk diffusion and MIC determination	EO solutions prepared at 90% (*v*/*v*), using acetone	7 μL of EO solution placed on each disk	EO showed higher antimicrobial activity against ETEC, no activity shown against beneficial *Lactobacillus* sp.	[22]
Lemon	*B. subtilis*, *E. coli*, *S. enterica* serovar Typhimurium, *S. aureus*	Disk diffusion method	-	0.1 mL of EO solution placed on each disk	Ripened lemon peel EO was more effective against all four strains than the unripe lemon peel EO.	[71]
Sweet orange	*Bacillus* sp., *E. coli*, *S. aureus*	Agar well diffusion method	Hot ethanol, Cold ethanol, Hot aqueous, Cold aqueous	50 and 100 μL of each extract placed on disk	Hot ethanolic extract (100 μL) most effective, showing inhibitory zone of 16, 15 and 16 mm against *Bacillus sp.*, *E. coli* and *S. aureus*, respectively.	[56]
Feijoa	*E. coli*, *S. aureus*	Agar well diffusion method	Water and Methanol extracts	100 μL of each extract placed on disk	Methanol extract was more effective (Inhibition zone for *E. coli* and *S. aureus* was 14.7 and 26.5 mm, respectively).	[65]
Sweet orange	*S. aureus*, *B. subtilis*, *E. coli*	MIC determination by tube dilution method	Light phase and cold-pressed EO	-	The MIC of light phase EO for *S. aureus*, *B. subtilis* and *E. coli* was 3.13, 1.56 and 0.78 μL/mL, respectively.	[37]
Sweet orange	*S. aureus*, *L. monocytogenes*, *P. aeruginosa*	MIC determination by agar dilution method	EO and hexane extracts	100 to 2.5 mg/mL	EO was effective against *L. monocytogenes* (MIC value of 15 mg/mL) but less active against *S. aureus* and *P. aeruginosa*. Hexane extract at 10 mg/mL concentration was most effective.	[24]
Sweet orange, Lime, Mandarin, Grapefruit	*B. subtilis*, *S. aureus*, *E. faecalis*, *E. coli*, *P. aeruginosa*, *N. gonorrhoeae*	Disk diffusion and MIC determination by agar dilution method	-	10 μL of EO solution placed on each disk	Lime peel was most effective. MIC of 14 and 11 μL/mL was recorded for *S. aureus* and *E. coli*, respectively.	[51]
Sour orange, Sweet orange, Grapefruit, Lemon	*S. aureus*, *E. coli*, *E. faecalis*, *B. cereus*	Agar well diffusion method	Aqueous extract	50 μL of 100 mg/mL of extract was dispensed in each well	The inhibition zones for *S. aureus*, *E. faecalis*, *B. cereus* and *E. coli* ranged from 10 to 18 mm, 9 to 17 mm, 11 to 18 mm, and 14 to 21 mm, respectively.	[55]
Bitter orange	*L. monocytogenes*, *S. aureus*, *E. coli DH5α*, *Citrobacter freundii*	Disk diffusion	Hexane extract	-	*S. aureus* was moderately sensitive to bitter orange extract (inhibition zone of 10mm). The extract did not inhibit Gram-negative organisms.	[52]
Grapefruit, Pummelo	*E. coli*, *P. aeruginosa*, *S. enterica* subsp., *S. aureus*, *E. faecalis*	Disk diffusion method	Cold-pressed and water-distilled extracted EO	100 μL of 10 and 20 mg/mL of EO was suspended in each well	20mg/mL of pummelo peel EO presented antimicrobial activity against Gram negative *Salmonella enterica* subsp. followed by *E. faecalis > E. coli* > *S. aureus* > *P. aeruginosa*.	[39]
Sweet orange, Sweet lemon, Lemon	*S. enterica* serovar Typhimurium, *E. coli*	Disk diffusion method	Hexane extract	-	The inhibitory zone for *S. enterica* serovar Typhimurium and *E. coli* ranged from 4 mm to 10 mm.	[53]
Pomegranate	*S. aureus*, *E. aerogenes*, *S. enterica* serovar Typhimurium and *K. pneumoniae*	Agar well diffusion method	Methanol, Ethanol (100, 70, 50, 30%), Water	10 μL of extract: water (1:6) was dispensed in each well	*S. aureus* was the most sensitive strain, followed by *E. aerogenes*, *S. enterica* serovar Typhimurium, *K. pneumoniae*. The inhibition zone for *S. aureus* ranged from 24.5 to 20.3 mm.	[69]
Banana	*S. aureus*, *S. pyogenes*, *Enterobacter aerogenes*, *K. pneumoniae*, *E. coli*, *Moraxella catarrhalis*	Agar well diffusion	Aqueous extract	-	*S. aureus* showed an inhibition zone of 30 mm, but *E. coli* was resistant to the extract.	[67]
*C. deliciosa*	*S. aureus*, *Micrococcus luteus*, *E. coli*, *P. vulgaris*	Agar diffusion method	-	15 μL of EO was dispensed on the agar surface	The inhibition zone for all tested organisms ranged from 8 mm to 30 mm.	[63]
Lemon, Sweet lemon	*S. aureus*, *S. epidermidis*, *S. agalactiae*, *E. faecalis*, *Streptococcus pneumoniae*, *S. pyogenes*, *E. coli*, *E. aerogenes*, *K. pneumoniae*, *Proteus sp.*, *S. enterica* serovar Typhimurium, *Acinetobacter sp.*, *Moraxella catarrhalis*, *P. aeruginosa*	Agar well diffusion method	Aqueous extract	20 μL of extract was dispensed in each well	The effect of lemon and sweet lemon peel on microbial isolates was not significantly different. The inhibition zone for lemon and sweet lemon ranged from 20–30 mm and 10–35 mm, respectively.	[62]
Grapefruit	*B. cereus*, *S. faecalis*, *E. coli*, *K. pneumoniae*, *Pseudo- coccus* sp., *S. enterica* serovar Typhimurium, *Shigella flexneri*, *S. aureus*	Agar well diffusion method	Methanol, Ethanol	100 μL of 8, 40 and 80 μg/mL concentrations of EO solutions were dispensed in each well	Methanol extract was more effective against all tested strains. *B. cereus* was the most sensitive bacteria (inhibition zone from 30.33 to 32.67 mm), while *E. faecalis* was the most resistant one (inhibition zone from 6.0 to 12.0 mm)	[72]
Pomegranate	*B. subtilis*, *S. aureus* *E. coli*, *K. pneumoniae*	Microdilution method	Methanolic and aqueous extracts	0.097–12.5 mg/mL	The MIC value for the tested strains ranged from 0.2 to 0.78 mg/mL.	[73]
Pummelo	*S. aureus*, *B. subtilis*, *E. coli*	Disk diffusion and MIC determination by broth microdilution method	-	10 μL of 50% (*v*/*v*) EO was placed on each disk. MIC concentration ranged from 1.17 to 750 μL/mL (*v*/*v*).	The inhibition zones for *B. subtilis*, *S. aureus* and *E. coli* were 17.08, 11.25 and 8.27 mm, respectively. The MIC values for *B. subtilis*, *S. aureus* and *E. coli* were 9.38, 9.38 and 37.50 μL/mL, respectively.	[40]
Pomegranate	16 strains of *Salmonella sp.*	Disk diffusion and MIC determination	Ethanol	20 μL of 100, 200 and 500 μg/mL concentration of EO solution was placed on each disk. MIC concentration ranged from 3.9 to 2000 μg/mL	The inhibition zone and the MIC values for *Salmonella sp.* ranged from 13.3 to 18.8 mm and 62.5 to 1000 μg/mL, respectively.	[74]
Lemon	*P. aeruginosa*, *S. enterica* serovar Typhimurium, and *Micrococcus aureus*	Agar well diffusion method and MIC determination	Methanol, Ethanol, Acetone	Dilutions from crude extract were prepared as follows: 1:20, 1:40, 1:60, 1:80, 1:100	All concentrations of lemon peel extracts effectively inhibited all the three strains tested.	[75]
Mandarin, Tangerine, Sweet orange, Lime, Grapefruit	*E. coli*, *S. enterica* serovar Typhi, *K. pneumoniae*, *E. cloacae*, *P. fluorescence*, *Proteus myxofaciens*, *S. epidermidis*, *Streptococcus* sp.	Disk diffusion method	-	From 500 μg/mL of stock solution 5 and 10 μL of EO was placed on each disk.	*S. enterica* serovar Typhi and *P. myxofaciens* were susceptible to all citrus EO tested.	[15]
Grapefruit	*S. aureus*, *E. faecalis*, *S. epidermidis*, *E. coli*, *S. enterica* serovar Typhimurium, *S. marcescens* and *P. vulgaris*	Disk diffusion method	-	20 μL of extract was dispensed in each well	*S. enterica* serovar Typhimurium was the most resistant (15 mm) strain followed by *E. faecalis* (16 mm), *S. epidermis* (17 mm), *S. marcescens* (19 mm), *P. vulgaris* (21 mm) and *S. aureus* (53 mm).	[38]
Pomegranate	*E. coli, Pseudomonas fluorescens, S. enterica* serovar Typhimurium, *S. aureus, B. cereus*	MIC determination by tube dilution method	Water	Final concentration of 0.01, 0.05, 0.1% was prepared in saline	*S. aureus* and *B. cereus* got inhibited at a concentration of 0.01%, *P. fluorescens* at 0.1%, *E. coli* and *S. enterica* serovar Typhimurium were not inhibited.	[76]
Pomegranate	*L. monocytogenes*, *S. aureus* *B. subtilis*, *E. coli*, *P. aeruginosa*, *K. pneumoniae*, *Yersinia enterocolitica*	Agar well diffusion and MIC determination by agar dilution method	Methanolic (80%) and water extracts	800 μg/100 μL of extract was suspended in each well. MIC concentration ranged from 0 to 4 mg/mL	The inhibition zone for methanolic extract ranged from 13–20 mm. MIC determination showed that *Y. enterocolitica* was the most sensitive strain representing MIC of 0.25 mg/mL.	[77]
Sour lime	*B. subtilis*, *B. cereus*, *S. aureus*, *E. coli*, *E. aerogenes S. enterica* serovar Typhimurium	Disk diffusion method	-	-	*B. subtilis*, *B. cereus*, *S. aureus*, *S. enterica* serovar Typhimurium, *E. coli* and *E. aerogenes* showed inhibition zones of 22, 19.8, 18, 17, 16 and 10.5 mm, respectively.	[68]
Sweet orange	*S. aureus*, *B. subtilis*, *E. coli*	Disk diffusion and MIC determination by broth microdilution method	-	10 μL of 50% (*v*/*v*) EO was placed on each disk. MIC concentration ranged from 1.17 to 750 μL/mL (*v*/*v*).	The inhibition zones for *S. aureus*, *B. subtilis* and *E. coli* were 23.37, 18.89 and 17.21 mm, respectively. The MIC values for *S. aureus*, *B. subtilis* and *E. coli* were 4.66, 9.33 and 18.75 μL/mL, respectively.	[54]
Lemon, Grapefruit, Bitter orange, Sweet orange, Mandarin, Bergamot	*S. aureus*, *B. cereus*, *Mycobacterium smegmatis*, *L. monocytogenes*, *M. luteus*, *E. coli*, *K. pneumoniae*, *P. aeruginosa*, *P. vulgaris*	Disk diffusion method	-	20 μL of EO solution was placed on each disk	Lemon peel EO exhibited better antimicrobial activity towards all bacteria with inhibition zone ranging from 10 to 16 mm.	[57]
Bergamot	*E. coli*, *P. putida*, *S. enterica*, *L. innocua*, *B. subtilis*, *S. aureus*, *Lactococcus lactis*	MIC determined using Bioscreen C	Ethanol (70, 100%)	200–1000 μg/mL	The MIC values for *E. coli*, *S. enterica*, *P. putida* were 200, 400, 500 μg/mL, respectively. Gram-positive bacteria showed no effect.	[61]

## 4. Effect of Chemical Components of Essential Oils on Food Spoilage and Pathogenic Microbes

**Figure 2:**
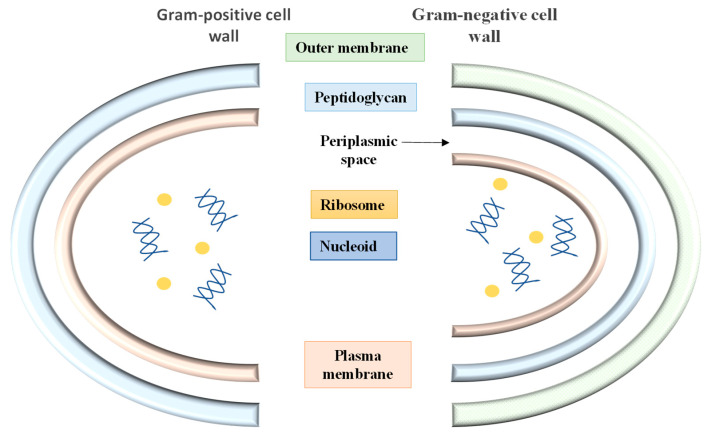
Schematic representation of Gram-positive and Gram-negative bacterial cell wall.

In the literature, various modes of antimicrobial activity of EOs against a range of bacteria have been discussed [5,7,78,79]. However, before investigating the effect of fruit peel EO on microbes, we should have a closer look at the cell-wall structure of Gram-negative and Gram-positive bacteria (Figure 2).

The hypothesis that Gram-positive bacteria are more susceptible to the effect of hydrophobic compounds such as EOs was first proposed by Plesiat et al. [80] followed by Nazzaro et al. [34], Chouhan et al. [79] and Raut et al. [81]. The difference between the susceptibility is attributable to the fact that Gram-positive bacteria have a thick layer of peptidoglycan linked to other hydrophobic molecules such as proteins and teichoic acid. This hydrophobic layer surrounding the Gram-positive bacterial cell may facilitate easy entry of hydrophobic molecules. On the other hand, Gram-negative bacteria have a more complex cell envelope comprising an outer membrane linked to the inner peptidoglycan layer via lipoproteins. The outer membrane contains proteins and lipopolysaccharides (lipid A), making it more resistant to the hydrophobic molecules in EO [82].

**Figure 3:**
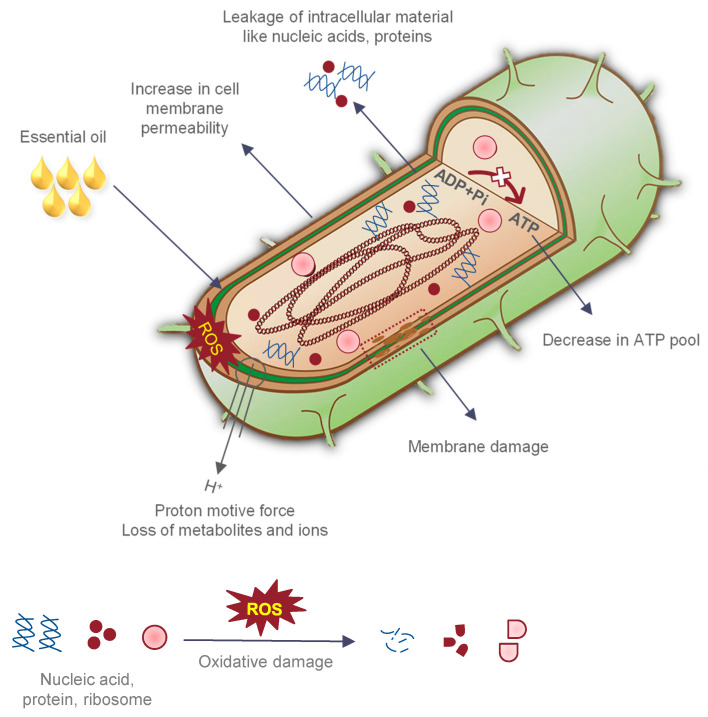
Antibacterial mechanism of essential oils (EOs).

Other researchers investigating the antimicrobial activity of EOs showed no notable difference between the MIC values of Gram-positive and Gram-negative bacteria [13,39,60,61,64]. Although it has been hypothesized that the outer membrane is almost impermeable to the hydrophobic compounds, Plesiat et al. [80] argued that some hydrophobic compounds might cross the outer membrane via porin channels. Similarly, Van de Vel et al. [58] believe that some EO molecules are more active against Gram-positive bacteria, while others are active against Gram-negative bacteria, but the mechanisms remain unknown. Most studies on the antimicrobial activity of EOs have used *E. coli* and *S. aureus* as model microorganisms to represent Gram-negative and Gram-positive bacteria, respectively [65,83,84]. This could lead to a generalization of results, as not all Gram-negative and Gram-positive bacteria would follow a similar trend as observed in *E. coli* and *S. aureus*. Furthermore, the mode of action of EO depends on its chemical profile and the ratio of its active components [85]. The possible mechanisms wherein EOs interfere with bacterial proliferation may involve the following: (1) the disintegration of the bacterial outer membrane or phospholipid bilayer, (2) alteration of the fatty acid composition, (3) increase in membrane fluidity resulting in leakage of potassium ions and protons; (4) interference with glucose uptake, and (5) inhibition of enzyme activity or cell lysis (Figure 3) [5,86].

In general, fruit peel EOs may comprise more than a hundred compounds [43]. Major compounds can contribute around 85–95% of the total EO composition, while other minor compounds can be present in trace amounts. While these compounds may have specific antimicrobial effects, Cho et al.’s [86] review draws attention to the synergistic and additive effect minor compounds might have in combination with the other components. Terpenes and terpenoids are primary components of essential oil followed by polyphenols [32]. Here, we discuss the antimicrobial activity and mode of action of EOs and their components on the bacterial cell.

### 4.1. Terpenes and Terpenoids

Terpenes and terpenoids constitute a significant class of compounds in EOs known to have antimicrobial activity. The potential antimicrobial activity of thymol and carvacrol has been extensively discussed in previous reviews [7,34,79]; hence we exclude them from our discussion to focus on other EO compounds. Thymol and carvacrol are the major components of thyme and oregano oil, respectively, and are structurally analogous differing in the location of hydroxyl groups on the phenol ring [7].

It is well recognized that terpenes can disrupt the lipid assembly of the bacterial cell wall, leading to disintegration of the cell membrane, denaturation of cell proteins, leakage of cytoplasmic material, which ultimately causes cell lysis and cell death [47]. Kim et al. [87] were amongst the first to show the antimicrobial potential of EO components including citral, limonene, perillaldehyde, geraniol, linalool, α-terpineol, carvacrol, citronellal, eugenol, β-ionone and nerolidol against *E. coli*, *S. enterica* serovar Typhimurium, *L. monocytogenes* and *Vibrio vulnificus*. It was suggested that terpenes and terpenoids might interfere with oxidative phosphorylation or oxygen uptake in microbial cells, thereby inhibiting microbial growth [88]. Later, this hypothesis was supported by Zengin and Baysal’s study [89], wherein terpene compounds such as linalool, α-terpineol and eucalyptol were reported to damage the cell membrane and alter the morphological structure of *S. aureus*, *S. enterica* serovar Typhimurium and *E. coli* O157:H7. The plausible explanation for this observation was that these terpene compounds interacted with the membrane proteins and phospholipids, leading to cellular respiratory chain inhibition, interruption in oxidative phosphorylation, disruption of nucleic acid synthesis, and loss of metabolites [90].

Two studies conducted by Togashi et al. [90,91] examined the effect of geranylgeraniol, geraniol, nerolidol, linalool and farnesol on *S. aureus*. All these terpene alcohols were reported to have antibacterial activity, with farnesol and nerolidol demonstrating the most potent antibacterial activity as determined by the broth dilution technique. They also explored the mechanism of these terpene alcohols on the bacterial cell membrane by measuring the leakage of K^+^ ions from the bacterial cell, anticipating that distortion of the bacterial cell membrane leads to leakage of K^+^ ions, thus indicating the presence of membrane disrupting compounds. In support of this, Akiyama et al. [92] reported the strong inhibitory effect of farnesol against *S. aureus*. Farnesol has also exhibited notable antibacterial activity against biofilms of *S. aureus* and *S. epidermidis* [93,94]. Akiyama et al. [92] attributed these inhibitory effects of farnesol to its hydrophobic nature, which accumulates in the cell membrane, thus disrupting the cell membrane as illustrated by scanning electron microscopy (SEM). Furthermore, an ester compound of geranyl acetate makes it a more potent antimicrobial compound than its parent moiety (geraniol), purportedly due to its hydrophobicity [95]. However, past studies [31,96] have demonstrated the antimicrobial activity and mechanism of geraniol, rather than geranyl acetate. For instance, geraniol was noted to inhibit *E. coli* and *S. aureus* [97], and multidrug-resistant *Enterobacter aerogenes* by acting as an efflux pump inhibitor [96,98]. Similar, to farnesol, it is thought that the antimicrobial potential of geraniol was due to its hydrophobic nature.

Han et al. [44] and Liu et al. [99] examined the antibacterial mechanism of limonene on *L. monocytogenes* and the antibacterial mechanism of linalool on *P. aeruginosa*, respectively. In their analysis, Han et al. [44] and Liu et al. [99] demonstrated that the compounds distorted the cell wall structure of bacteria and led to leakage of intracellular molecules such as nucleic acids and proteins, which also affected the functionality of the respiratory chain complexes and hampered the process of adenosine triphosphate (ATP) synthesis. Moreover, Gao et al. [100] elaborated the anti-listeria activities of linalool against its planktonic cells and biofilms using RNA-sequence analysis. Other articles have discussed the antimicrobial efficacy of limonene [101] and linalool [102] against various strains of microorganisms. The antimicrobial activity of limonene is due to the presence of alkenyl substituent and a double bond in the molecular structure that enhances its antimicrobial activity [95]. Other authors proposed that the cell membrane may be an important site for linalool to inactivate the cell [100]. The interaction causes thickening of the Gram-positive cell wall, eventually leading to cell disruption [103]. The *S* (+) enantiomer of linalool enables it to interact with the negatively charged outer membrane of the Gram-negative cell, thus facilitating the easy entry of the compound into the intracellular space, leading to disruption [104].

Dorman et al. [105] tested 14 EO compounds against 25 strains of bacteria and reported that monoterpenoid and sesquiterpene demonstrate potent antimicrobial activity against most strains tested. In the same way, Trombetta et al. highlighted the antimicrobial potential of monoterpenes (linalyl acetate, thymol and menthol) against *E. coli* and *S. aureus* [106]. The hydroxyl group present in the compound may have contributed to its antimicrobial activity. Guimaraes et al. [31] evaluated 33 terpene compounds commonly isolated from EOs for their antimicrobial efficacy, of which only 16 compounds were reported to possess antibacterial activity. Scanning electron microscopy results revealed that individual components of EOs such as geraniol, citronellol, carveol, and terpineol altered the cellular morphology and destroyed the cell membrane. This is supported by two previous studies where similar compounds were found to be potent [105,106]. Lopez-Romero et al. [107] conducted a similar study wherein the antibacterial effect and mechanism of action of essential oil components such as carveol, carvone, citronellol, and citronellal were evaluated against *E. coli* and *S. aureus*. Citronellol was found to be the most effective, which led to a change in the cell membrane integrity, the surface charge followed by leakage of K^+^ ions. In another study, two pentacyclic triterpenes, namely α-amyrin and ursolic acid, were also reported to have a disorganizing effect on the *E. coli* cell membrane [108]. Additionally, Garcia et al. [66] listed five monoterpene compounds (citronellal, citral, α-pinene, isopullegol and L-carvone) which possessed antifungal properties against three fungal strains and suggested their potential use in tropical fruit preservation. Other researchers [109,110] have investigated the antimicrobial potential of a bicyclic sesquiterpene, i.e., β-caryophyllene, against a range of microorganisms. However, they were unable to explain for the antibacterial mechanism with their study.

### 4.2. Polyphenols

Studies on polyphenols extracted from various fruit sources are well represented in the literature, and it is acknowledged that polyphenols possess a range of antimicrobial activities against pathogenic microbes. For example, the polyphenols in the skin extracts of Italian red grape, plum and elderberries demonstrated strong inhibitory properties against *S. aureus*, *B. cereus*, *E. coli*, *L. monocytogenes* while showing a growth-promoting effect on beneficial microbes such as *Lactobacillus rhamnosus*, *L paracasei* and *Lactobacillus plantarum* [111].

#### 4.2.1. Phenylpropenes

Although phenylpropenes account for a smaller proportion of total volatiles than terpenes and terpenoids, they have been noted to have a significant contribution to the antimicrobial activity of EOs [112]. Phenylpropenes are not only found in some fruit varieties such as apple peel [113], lemon peel [114] and grapefruit peel [115], but are also found in a wide variety of spices and herbs such as clove, star anise, sweet basil and fennel [116].

The antimicrobial potential of eugenol has been extensively investigated [117,118,119,120]. Eugenol is thought to alter the permeability of the cell membrane, followed by leakage of intracellular ATP and macromolecules such as protein and nucleic acids, ultimately leading to cell death [119]. This theory was supported by Cui et al.’s [118] study wherein eugenol permeabilized the cell membrane leading to leakage of intracellular macromolecules and enzymes such as β-galactosidase, ATP and alkaline phosphatase (AKP). Furthermore, Qian et al. [117] noted that eugenol demonstrates cell membrane permeability properties and presents potent inhibition against the biofilm formation of *K. pneumoniae* cells. Likewise, Ashrafudoulla et al. [119] reported antibiofilm activity against *Vibrio parahaemolyticus* and cell membrane damaging properties, which led to leakage of cell contents. Research by Nazzaro et al. found that isoeugenol worked in a similar way to eugenol [34]. Hyldgaard et al. [121] explained that isoeugenol formed hydrogen bonds with the lipid headgroup, thus disturbing the lipid structure and destabilizing the membrane. This mechanism of action is known as a “non-disruptive detergent-like mechanism”, and the free hydroxyl group and the molecule’s hydrophobic nature were considered accountable for their antimicrobial activity [122]. However, Gharib et al. [112] argued that hydrophobicity might not be the only factor contributing to the molecule’s antimicrobial activity, since in his study, eugenol and isoeugenol demonstrated a fluidizing effect on the bacterial cell wall. Furthermore, Auezova et al. [123] and Gharib et al. [112] examined the mechanism of allylic (eugenol and isoeugenol) and propenylic (estragole and anethole) phenylpropenes on the cell wall of *E. coli* and *Staphylococcus* *epidermidis*. They demonstrated the distinctive ability of estragole and anethole to penetrate the outer membrane of *E. coli*. The antimicrobial potency is conferred by the higher lipophilic nature of estragole and anethole (log *P* values of 3.5 and 3.4, respectively) in comparison to eugenol and isoeugenol (log *P* values of 2.5 and 3.0, respectively).

Cinnamaldehyde has also demonstrated anti-biofilm activities against *S. epidermidis* [124]. Other researchers have studied the antibacterial mechanism of cinnamaldehyde against *E. coli*, *S. aureus* [125] and *Aeromonas hydrophila* [126], reporting that it caused cell membrane distortion and leakage, in addition to condensation and polarization of the cytoplasmic content. The antibacterial activity of vanillin was studied against *Mycobacterium smegmatis,* and it was able to enhance the cell membrane permeability and alter cell membrane integrity [127].

#### 4.2.2. Flavonoids

Flavonoids are polyphenolic compounds with a benzo-γ-pyrone group and are ubiquitously found in plant cells [36]. Few examples of flavonoids are flavanones, flavan-3,4-diols, chalcones, flavan-3-ols, flavonols, flavones, isoflavones, catechins, quercetin, anthocyanidins and proanthocyanidins [128]. Recent evidence suggests that flavonoids possess antibacterial activities against plant pathogens and human pathogens. Their antimicrobial mechanism is similar to traditional drugs [33], and hence could be of importance for use as natural antimicrobial agents.

A study on catechins showed that the compounds caused oxidative damage in *E. coli* and *B. subtilis* cells, thus altering cell membrane permeability and damaging the cell membrane [129]. Moreover, Cushnie et al. [130] also reported that catechins were responsible for potassium ion leakage in methicillin-resistant *S. aureus* (MRSA), which is the primary signal of membrane damage, and Tsuchiya et al. [131] reported that sophoraflavanone G significantly affected the membrane fluidity of the bacterial cells.

## 5. Application of Essential Oils in Food Products

### Preservation

Traditional food preservation methods include chilling, frozen storage, drying, salting, smoking and fermentation [132]. However, consumers have questioned techniques such as fermentation, brining, and salting, due to the increasing demand for reduced-salt foods [133]. The meat industries utilize chemical preservatives such as nitrate salt, sulfites, chlorides to inhibit the growth of foodborne pathogens. These compounds have been associated with carcinogenic effects and other health complications [133]. Hence, the options available to substitute chemical preservatives with natural compounds have attracted increased interest in recent years. Lucera et al. [134], in her review, outlined some natural preservatives of animal origin, (lactoferrin, lysozyme); bacteriocin from microbes (natamycin, nisin); natural polymers (chitosan); organic acids (citric and propionic acid); EOs and extracts derived from plants. In this context, EOs are attracting considerable attention due to their application as a natural bio-preservative and inhibitor in food matrices or food products. At present, the investigations have focused primarily on EOs from herbs and spices. There is limited research on fruit peel EOs. So, the discussion is widened here to cover the food applications of all plant-derived EOs. Some publications have investigated the potential contributions EOs/extracts to extend the shelf-life and to inhibit the growth of pathogens in fresh-cut vegetable mixtures [135], lettuce, purslane [136], fruit juices [137], ready to eat meat [138], chicken nuggets [76] and breast [139], minced beef [140,141] and turkey [142]. A literature review [141] published in 2018 included 2473 publications since 1990 on the antimicrobial activity of EOs. Many of these publications investigated the application of EO’s on food products, including 657 papers on fruits, 403 on vegetables, 415 on fish products, 410 on meat products, 216 on milk and dairy products, and 97 on bread and baked foods [143]. Other recent reviews have discussed the application of rosemary extract in meat [144], the synergistic effect of EO in seafood preservation [145], application of EO in active packaging [146] and as a food preservative [147]. The following section includes the recent history of EO by restricting the citations to the last 5–6 years to provide the readers with an update on EOs and their application in the food matrix (Table 2).

As consumers have gained greater awareness on issues related to health, processing and food additives, demand for natural and minimally processed food has soared. However, maintaining the freshness of fruits and fresh-cut vegetables for extended periods has been challenging. Spraying, dipping, coating, and impregnation are ways EOs can be applied to fruits and vegetables for maintaining shelf-life [134]. Some recent examples of these approaches are discussed here. He et al. [148] evaluated the effects of dipping cherry tomatoes in thyme EO nanoemulsion (TEON) against *E. coli* O157:H7 and the effect of TEON in combination with ultrasound treatment. Their study showed that TEON alone could effectively inhibit the growth of *E. coli* O157:H7 on the surface of cherry tomatoes, and there was a substantial synergistic effect of the combined treatment. Kang et al. [149] found that freshly cut red mustard leaves, when washed with cinnamon leaf EO nanoemulsion, reduced the count of *E. coli*, *L. monocytogenes*, *S. enterica* serovar Typhimurium by more than one log. Another study conducted by the same author showed that washing with cinnamon leaf EO nanoemulsion improved physical detachment and inhibited both *L. monocytogenes* and *E. coli* O157:H7 on kale leaves [150]. Both studies did not show any adverse changes in the quality attributes of mustard [149] and kale leaves [150]. The lettuce leaves examined during 7-day storage periods showed a reduction in *E. coli* O157:H7 population when rinsed with a combination of carvacrol/eugenol and thymol/eugenol when compared to the control (water rinse). However, the treatments had adverse effects on the sensory analyses [151]. In contrast, a combination of Spanish origanum oil and Spanish marjoram oil successfully inhibited *L. monocytogenes* from a mixture of fresh-cut vegetables without showing any adverse sensory attributes [135]. A recent study elucidated that *Litsea cubeba* EO added to bitter gourd, cucumber, carrot and spinach juices at MIC concentration decreased the counts of *E. coli* O157:H7 by 99.1%, 99.92%, 99.94%, 99.96%, respectively [152]. Krogsgård Nielsen et al. [153] tested the inhibitory potential of isoeugenol and encapsulated isoeugenol against *L. monocytogenes*, *S. aureus*, *Leuconostoc mesenteroides*, *P. fluorescens* in carrot juice. Contrary to expectations, their study did not find a significant difference in the inhibitory activity of encapsulated and non-encapsulated isoeugenol.

Besides fruits and vegetables, much work on the antimicrobial potential of EO was studied in meat products especially beef and beef products [154,155,156,157,158]. Pistachio EO [155] and *Melaleuca alternifolia* (tea tree) EO [157] reduced the total viable and total *L. monocytogenes* counts in ground beef. The efficiency of 5% and 10% clove EO on the inactivation of *L. monocytogenes* in ground beef at refrigeration (8 °C), chilling (0 °C) and freezing (18 °C) temperatures was investigated by Khaleque et al. [158]. They observed that 10% clove EO was a lethal concentration to inactivate *L. monocytogenes* irrespective of temperature conditions, but 5% clove EO was ineffective at inactivating the pathogen [158]. Similarly, Yoo et al. [154] found that 0.5%, 1.0% and 1.5% clove EO did not significantly reduce the count of *E. coli* O157:H7 and *S. aureus* in beef jerkies. However, their study took an additional step and demonstrated that the combined effect of clove EO with encapsulated atmospheric pressure plasma had a bactericidal effect on both pathogens. Likewise, a study conducted by Lin et al. [156] pointed out the synergistic effect of chrysanthemum EO incorporated into chitosan nanofibers which inhibited *L. monocytogenes* in beef at a rate of 99.9%.

A triple combination of thyme/cinnamon/clove EO in the food matrix was first applied experimentally by Chaichi et al. [159]. The triple combination at FIC of 0.3, 0.39.0.43 had a bacteriostatic effect on *P. fluorescens* inoculated in chicken breast meat, while a triple combination at higher concentration (200 mg/kg) had an instant bactericidal effect. Thyme EO effectively inhibited *P. aeruginosa*, *E. coli* and *S. enterica* serovar Typhimurium in ground beef [160]. A recent study by Kazemeini et al. [161] prepared edible coatings of alginate containing *Trachyspermum ammi* EO (TAEO) as nanoemulsion to control the growth of *L. monocytogenes* in turkey fillets. The turkey fillets were coated with the emulsion and stored at 4 °C for 12 days. They observed the highest reduction of *L. monocytogenes* numbers in turkey fillets treated with 3% alginate containing 0.5% and 1% TAEO compared to non-coated samples. Other research articles have reported that EO nano emulsions effectively inhibited pathogens in rainbow trout fillet [162] and chicken breast fillets [163]. Apart from fruits, vegetables and meat products, the application of EO has also been evaluated on bakery [164,165] and dairy products [166].

Although several authors [152,157,165,166] have claimed successful testing for the application of EOs in different food systems, their approach has not escaped criticism. Santos et al. [167] emphasized the use of MBC concentration rather than MIC concentration in the food matrix to ensure a complete inhibition. These authors [167] questioned the usefulness of EOs in food systems because various factors such as environmental condition, age and cultivar of the plant, time harvested, extract composition and extraction method may impact the antimicrobial activity of the EO. All the above factors might challenge the rationale of applying EOs at a commercial level. Moreover, it is known that fat and protein present in food can solubilize or bind to phenolic compounds in EO, thus reducing its antimicrobial efficacy [157]. This view was supported by Khaleque et al. [158], who analyzed the effect of cinnamon EO at a higher concentration (2.5 and 5.0%) against *L. monocytogenes* in ground beef and found that cinnamon EO was unsuccessful in inactivating *L. monocytogenes* in ground beef. They also reported adverse organoleptic impacts upon using higher concentrations of EOs. In a study by Lages et al. [168], thyme EO combined with beet juice powder failed to give a desirable effect in reducing coagulase-positive *Staphylococcus* in meat sausage. It was recommended that combining half of the suggested dosage of chemical preservatives such as nitrites with EO could be feasible. Despite the question regarding the suitability of EO in minimally processed food products [167], only a few studies did not show effective inhibition by EOs of foodborne pathogens. In contrast, many studies have demonstrated the successful replacement of synthetic preservatives with EO in different food systems [165,166,169]. Since Santos et al. [167] did not use EOs in minimally processed food products, their assumptions need further validation. Their paper would have been more convincing if the authors had used food matrices to prove their hypothesis. There is evidence that EOs exhibit antimicrobial properties, therefore, their ability to be used as a natural preservative on an industrial scale needs further rigorous evaluation.

**Table 2:** Overview of recent studies on antimicrobial activity of different essential oils (EOs) in the food matrix.

Essential Oil	Pathogen	Food	Method Used	Concentration Applied	References
Thyme (*Thymus vulgaris*)	*E. coli* O157:H7	Cherry tomatoes	Dipping	0.0625, 0.125 mg/mL	[148]
Clove (*Syzygium aromaticum*)	*E. coli* O157:H7, *S. aureus*	Beef jerkies	Treated with EO and dried for 2 hrs	0.50%, 1.00%, 1.50%	[154]
Ajwain (*Trachyspermum ammi*)	*L. monocytogenes*	Turkey fillets	Coating	8, 4, 2 mg/mL	[161]
May chang (*Litsea cubeba*)	*E. coli* O157:H7	Bitter gourd, cucumber, carrot, and spinach juice	Inoculation	0.5, 0.25 mg/mL	[152]
Felon herb (*Artemisia persica* Boiss)	*L. monocytogenes*, *E. coli* O157:H7	Probiotic doogh	Addition of EO and mixing	75 ppm, 150 ppm	[164]
Rosemary (*Rosmarinus officinalis*), Lavender (*Lavandula*), Mint (*Mentha piperita*)	*Penicillium crustosum*	Bread	Exposing bread to a disk loaded with EO	125, 250, 500 µL/L	[165]
Thyme (Thy), Cinnamon (CN) (*Cinnamomum verum*), Clove (CV)	*P. fluorescens*	Chicken breast	Coated by dipping in EO emulsion for 5 min	Thy- 0.560 g/L, CN- 0.042, 0.170 g/L, CV- 0.078, 0.312 g/L	[159]
Ginger (*Zingiber officinale*), Clove, Thyme	*S. aureus*, *P. aeruginosa*, *E. coli*, *E. faecalis*, *P. fluorescens*, *C. albicans* and *Aspergillus parasiticus*	Fortified cheese	EO added and stirred	0.01%	[166]
Tea tree (*Melaleuca alternifolia*)	TVC, Psychrophilic, Coliform, Salmonella, Yeast, and mould count	Beef steaks	Addition of EO and mixing	0.1%, 0.5%	[155]
Cranberry extract (*Vaccinium macrocarpon*)	*Listeria* sp.	Chicken breast	Dipped in extract solution	4, 8 mg/mL	[170]
Thyme	Thermotolerant coliforms and *Escherichia coli*	Hamburger	Addition of EO and mixing	0.1 g/100 g of thyme EO 1 g/100 g of encapsulated thyme EO	[169]
Cinnamon leaf EO nanoemulsion	*L. monocytogenes*, *E. coli* O157:H7	Kale leaves	Washing	50 ppm	[150]
Thymol, Eugenol, Carvacrol	*E. coli* O157:H7	Lettuce leaves	Rinsing	0.63 mg/mL	[151]
Chrysanthemum (*Chrysanthemum indicum*)	*L. monocytogenes*	Beef	Packed into membrane (Chitosan nanofiber loaded with EO)	1.5%	[156]
Pistachio (*Pistacia vera*)	Total viable count (TVC)	Ground beef	EO added to meat and stomached for 1 min	1.5% (*v*/*w*)	[157]
Black cumin (*Bunium persicum*)	*E. coli* O157:H7	Rainbow trout fillet	Coated by dipping in nanoemulsion for 15 min	0.5%	[162]
Cranberry extract (*Vaccinium macrocarpon*)	Aerobic mesophilic count, *Brochothrix thermosphacta*, *P. putida*, *L. mesenteroides*, *L. monocytogenes*, *C. jejuni*	Pork meat slurry, hamburger, cooked ham	Mixed in meat	3.3%, 1.65%, 0.83%, 0.42%	[171]
Anise (*Pimpinella anisum*)	TVC, Psychotropic count, Enterobacteriaceae, Lactic acid bacteria, *Pseudomonas* sp.	Minced beef	EO added using micropipette and massaged manually for 2min	0.1%, 0.3%, 0.5% (*v*/*w*)	[172]
Coriander (*Coriandrum sativum*)	TVC, sulphite-reducing clostridia, *Salmonella* sp., *E. coli*, *L. monocytogenes*	Pork sausage	Mixed in sausage	0.000, 0.075, 0.100, 0.125, 0.150 μL/g	[173]
Cinnamon	TVC, Enterobacteriaceae	Italian pork sausage	Mixed in sausage	0.1%, 0.5% (v/m)	[174]
Ginger	Psychrophilic, Yeast and mould count	Chicken breast fillet	Coated by dipping in emulsion	3%, 6%	[163]
Cranberry extract	*E. coli*, *Salmonella enterica* serovar Enteritidis, *L. monocytogenes*, *S. aureus*	Minced pork		2.5 g/100 g	[175]
Thyme	*E. coli*, *S. enterica serovar Typhimurium*, *S. aureus*, and *P. aeruginosa*	Minced beef meat	EO added to meat and stomached for 5 min	0.001%, 0.05%, 3% of EO in 10% DMSO (*v*/*w*)	[160]
Cinnamon EO (CEO) and grape seed extract (GSE)	TVC, Lactic acid bacteria, Psychotropic count, Yeast, and mould count	Sausage	Mixed in sausage and packed in polyamide bags	CEO (0.02% and 0.04%) and GSE (0.08% and 0.16%)	[176]
Apple mint (*Mentha suaveolens*)	*E. coli*, *S. aureus*	Turkey sausage		2, 5, 10 mg/g	[177]
Isoeugenol	*L. monocytogenes*, *S. aureus*, *Leuconostoc mesenteroides*, *P. fluorescens*	Carrot juice	Inoculation	702, 1580 mg/mL	[153]
Thyme	*Salmonella enterica* serovar Enteritidis, *S. enterica serovar Typhimurium,* *S.* Montevideo and *S.* Infantis	Minced pork	Mixed in minced meat and vacuum packed	0.3%, 0.6%, 0.9%	[178]
Thyme	*L. monocytogenes*	Beef and pork sausage	Mixed and vacuum packed	100 ppm	[179]
Clove, Cinnamon	*L. monocytogenes*	Ground beef	Adding and mixing	Clove—5%, 10% Cinnamon—2.5%, 5%	[158]
Spanish origanum oil, Spanish marjoram oil and coriander oil	*L. monocytogenes*	Fresh cut vegetables	Immersing in EO solution	0.1%, 0.4%, 0.9%	[135]
Peppermint (*Mentha piperita)*	*Vibrio* spp.	Cheese	Applying on surface	5–15 µL/mL	[180]

## 6. Food Regulations on Applications of Essential Oils

The European Commission has documented a variety of EO compounds as approved flavour additives in different types of food products. In 2008, the European Commission released a list of approved compounds which is updated regularly. Some of the registered flavoring compounds that pose no risk to human health are limonene, linalool, β-caryophyllene, pinene, thymol, carvacrol, carvone, eugenol, isoeugenol, vanillin, citral, citronellal, cinnamaldehyde, menthol and lavandulol [181]. Moreover, the Food and Drug Administration (FDA) of the United States also recognizes these compounds as GRAS. Crude EOs such as mustard, oregano, clove, cinnamon, nutmeg, thyme, basil, rosemary and lavender are recognized as GRAS. The regulatory limits on acceptable daily intake on EO compounds and EOs are in place to govern their use in food products [7]. Despite the regulatory limits, EOs might cause allergic reactions and ingesting high doses of EOs or topical applications of EOs for a long period have been associated with severe health problems, such as oral toxicity and dermatitis [182]. Therefore, it is crucial to attain a fine balance between toxicity and effective dose in food products.

## 7. Conclusions and Future Prospects

Evidence from in vitro and in situ studies suggests that EOs possess good antibacterial activity against a wide range of foodborne pathogens. This review has evaluated studies on EOs that have the potential to act as natural preservatives in food products, due to their antioxidant and antimicrobial properties [183,184]. The potential of all plant-derived EOs, not just fruit peel EOs, has been evaluated for use as a preservative in foods. However, their application in food products have been restricted at an industrial scale as high doses are required to attain good antimicrobial activity, and the quantity, source and active composition profile of the EO to be used in food has not been optimized. In addition, components of the foods, such as fat [185], starch [186] and protein [187], may bind to the active compounds in EOs and reduce their efficacy. The volatile compounds in EOs may also produce undesirable chemical compounds by interacting with other food components such as proteins. To validate the use of EOs at an industrial level, the evaluation of these aspects is of paramount importance.

Firstly, high concentrations of EO in food have shown unappealing sensory attributes. However, this problem may be addressed by evaluating an effective synergistic/additive combination of EOs or a combination of EOs with other food preservation techniques such as temperature, irradiation, and pulse-electric field to reduce the required dosage of EO for the inhibition of pathogens. Another plausible solution for minimizing the interaction of EO compounds with food components such as fat, starch and proteins is by encapsulating the EO in an appropriate biodegradable material (e.g., chitosan), which might ensure controlled release without altering its biological activity. Secondly, a detailed understanding of how EOs work (the mechanism of action) will provide insights into the application of EO in the food industry to combat the proliferation of food-borne pathogens. To further study the mechanism of action, proteomic and transcriptomic analyses are needed to understand the pathways targeted by the EO compounds. The transition of in vitro experiments to in vivo trials to evaluate the efficacy of EOs has always posed an added challenge. Another future opportunity lies in the potential effects of EOs on immunity and gut health. Recent research reported that a combination of oregano extract with peppermint and thyme EO supported the growth of probiotic bacteria and positively affected the gut’s microbial composition [188]. Further research regarding the role of EO on the gut microbiome would be worth exploring.
